# Paleogenomics Reveals a Loss of Bovine Lineages in Mid-latitude Asia Over the Last 200,000 Years

**DOI:** 10.1093/gbe/evaf206

**Published:** 2025-11-06

**Authors:** Alexandre Gilardet, Jonas Oppenheimer, Mikkel-Holger S Sinding, Edana Lord, J Camilo Chacón-Duque, Gonzalo Oteo-García, Georgios Xenikoudakis, Pavel Kosintsev, John Southon, Sergey K Vasiliev, Michael V Shunkov, Maxim B Kozlikin, Katerina Douka, Beth Shapiro, Peter D Heintzman, Love Dalén

**Affiliations:** Centre for Palaeogenetics, Stockholm 10691, Sweden; Department of Zoology, Stockholm University, Stockholm 10691, Sweden; Department of Biomolecular Engineering, University of California Santa Cruz, Santa Cruz, CA 95064, USA; Department of Biology, The University of Copenhagen, Copenhagen 2100, Denmark; Centre for Palaeogenetics, Stockholm 10691, Sweden; Department of Zoology, Stockholm University, Stockholm 10691, Sweden; Centre for Palaeogenetics, Stockholm 10691, Sweden; Department of Zoology, Stockholm University, Stockholm 10691, Sweden; Centre for Palaeogenetics, Stockholm 10691, Sweden; Department of Archaeology and Classical Studies, Stockholm University, Stockholm 11418, Sweden; Centre for Palaeogenetics, Stockholm 10691, Sweden; Department of Archaeology and Classical Studies, Stockholm University, Stockholm 11418, Sweden; Institute of Plant and Animal Ecology, Urals Branch of the Russian Academy of Sciences, Yekaterinburg, Russia; Department of History, Ural Federal University, Ekaterinburg, Russia; Department of Earth System Science, University of California, Irvine, CA, USA; Institute of Archaeology and Ethnography, Siberian Branch, Russian Academy of Sciences, Novosibirsk, Russia; Institute of Archaeology and Ethnography, Siberian Branch, Russian Academy of Sciences, Novosibirsk, Russia; Institute of Archaeology and Ethnography, Siberian Branch, Russian Academy of Sciences, Novosibirsk, Russia; Department of Evolutionary Anthropology, Faculty of Life Sciences, University of Vienna, Vienna, Austria; Department of Biomolecular Engineering, University of California Santa Cruz, Santa Cruz, CA 95064, USA; Centre for Palaeogenetics, Stockholm 10691, Sweden; Department of Geological Sciences, Stockholm University, Stockholm 10691, Sweden; Centre for Palaeogenetics, Stockholm 10691, Sweden; Department of Zoology, Stockholm University, Stockholm 10691, Sweden; Department of Bioinformatics and Genetics, Swedish Museum of Natural History, Stockholm, Sweden

**Keywords:** ancient DNA, bovine, yak, Denisova Cave, Altai, Siberia

## Abstract

Bovines have a complex yet poorly understood evolutionary history that is characterized by admixture and diversity loss during the Late Pleistocene. Unraveling this history is challenging in part because deep-time and geographically widespread genetic data are currently limited. In mid-latitude Asia, Denisova Cave, located in the Altai, Siberia, and nearby paleontological sites have yielded a large collection of remains spanning the Middle to Late Pleistocene, many of which are identifiable as bovines via morphology or paleoproteomics. In this study, we screened these bovine bones for ancient DNA and generated mitogenomes, to refine knowledge of Pleistocene bovine diversity in the region. We found that bovines carrying a yak-like mitogenome were common residents of the Altai mountains, along with bison belonging to the clade X mitochondrial lineage and, more rarely, aurochs. The yak-like mitochondrial lineage identified in this study represents a previously unknown lineage sister to present-day yak mitogenome diversity. This yak-like mitochondrial lineage, termed yak X, was identified at several sites, and survived in mid-latitude Asia across climatic transitions for around 200,000 years. Our findings suggest that all three bovine taxa harbored diversity no longer present in extant populations, thus mirroring archaic hominin findings at Denisova Cave. The Altai mountains therefore appear to have been a hotspot of both bovine and hominin diversity.

Significance This study refines knowledge of bovine diversity in mid-latitude Asia throughout the Late and Middle Pleistocene, using ancient DNA from bone fragments. It circumvents challenges in taxonomic assignment encountered by other methods such as morphological identification, paleoproteomics, or sedimentary ancient DNA, and broadens our understanding of the largely understudied Middle Pleistocene. Denisova Cave, located in Siberia, became famous for the discovery of archaic hominins. We find that this site additionally harbored divergent and extinct lineages of bison X, aurochs, and bovines carrying a yak-like mitogenome, and thus appears to have been a hotspot of diversity lost to modern populations not only for hominins but also for bovines. The yak-like mitochondrial lineage we identified throughout mid-latitude Asia belongs to a previously unknown “X” lineage sister to, yet deeply divergent from, all known yak diversity.

## Introduction

Current understanding of bovine evolution is based primarily on the analysis of genomic data from modern-day and Late Pleistocene (ca. 129 to 12 ka, thousand years before present) individuals. However, wild extant and extinct bovines, such as yak (*Bos mutus*), bison (*Bison priscus, B. bonasus, B. bison*), aurochs (*Bos primigenius*), banteng (*Bos javanicus*), and gaur (*Bos gaurus*), have long evolutionary histories that span multiple glacial cycles of the Middle Pleistocene (ca. 780 to 129 ka) (Verkaar et al. 2004; Ho et al. 2008). The Bovini tribe probably originated in South Asia during the Late Miocene (before 5.3 Ma, million years before present) (Bibi 2007) before expanding into much of Eurasia and diversifying into multiple distinct lineages during the Pliocene (ca. 5.3 Ma to 2.6 Ma) and Early Pleistocene (ca. 2.6 Ma to 780 ka) (Hassanin et al. 2013).

Paleogenetic data spanning an extended time series into the Middle Pleistocene are crucial to understanding the richness and spatiotemporal distribution of these bovine lineages. However, well-dated paleontological and archaeological sites spanning genomic deep-time with sufficient DNA preservation to permit paleogenomic analysis are rare. One exception is Denisova Cave in the Altai mountains of southern Siberia. During the Pleistocene, the Altai mountains were located along an important south–north migration route for mammals and have been previously described as a contact zone for Eurasian faunal populations (Agadjanian and Shunkov 2018).

A finger bone fragment from Denisova Cave yielded a genome from an entirely new hominin lineage (Reich et al. 2010; Meyer et al. 2012). However, only a minute fraction of the remains excavated from Denisova are of hominin origin. The vast majority of bones excavated there are too fragmentary for morphological identification. To date, a collection of more than 15,000 of these bone fragments has been screened using a paleoproteomics method known as Zooarchaeology by Mass Spectrometry (ZooMS) (Buckley et al. 2009). For fragments with good biomolecular preservation, collagen profiles generated by ZooMS have allowed for taxonomic assignment to the genus level (Brown et al. 2021), and provided a more nuanced snapshot of mammalian diversity at Denisova Cave. A large fraction of the remains analyzed from Denisova using ZooMS were assigned to the bovine genera *Bos* and *Bison*.

Taxonomic profiles built using a combination of paleoproteomics, morphology, and sedimentary ancient DNA (sedaDNA) datasets have already revealed the presence of multiple bovine taxa in the Altai (Slon et al. 2017; Agadjanian and Shunkov 2018; Brown et al. 2021; Zavala et al. 2021; Jacobs et al. 2025). Morphological surveys of bovine specimens at Denisova Cave and multiple other sites of the broader Altai region have almost solely reported the presence of the steppe bison (*Bison priscus*) with a few yak occurrences (Vasiliev et al. 2017, 2018a, 2018b; Agadjanian and Shunkov 2018). However, morphological identification of bovine bones can be challenging, particularly from highly fragmented bone assemblages, and should thus be refined with genetic surveys. Targeted enrichment of mammalian mitogenomes from sedaDNA has instead shown a majority of American bison (*Bison bison*) and steppe bison (*Bison priscus*) hits, followed by the European bison (*Bison bonasus*) and yak (both domesticated *Bos grunniens* and wild *Bos mutus*) (Zavala et al. 2021; Jacobs et al. 2025). However, deconvoluting the species present in mixtures of closely related species can be challenging.

This study aimed to taxonomically characterize bovine assemblages from mid-latitude Asia using shotgun-sequenced ancient DNA (aDNA) from single bones, which circumvents the previously mentioned biases in morphological assignment and sedaDNA mitogenome capture. Furthermore, aDNA offers the possibility to resolve taxonomy to species and population levels, while paleoproteomics can only confidently assign family or genus level information. The sites included in this study, combined with evidence for a rich bovine presence across time and climatic stages, provide an ideal opportunity for investigating bovine diversity in a well-defined spatiotemporal context.

## Results

### Bovine Mitochondrial Diversity in Mid-latitude Asia

**Fig. 1.**
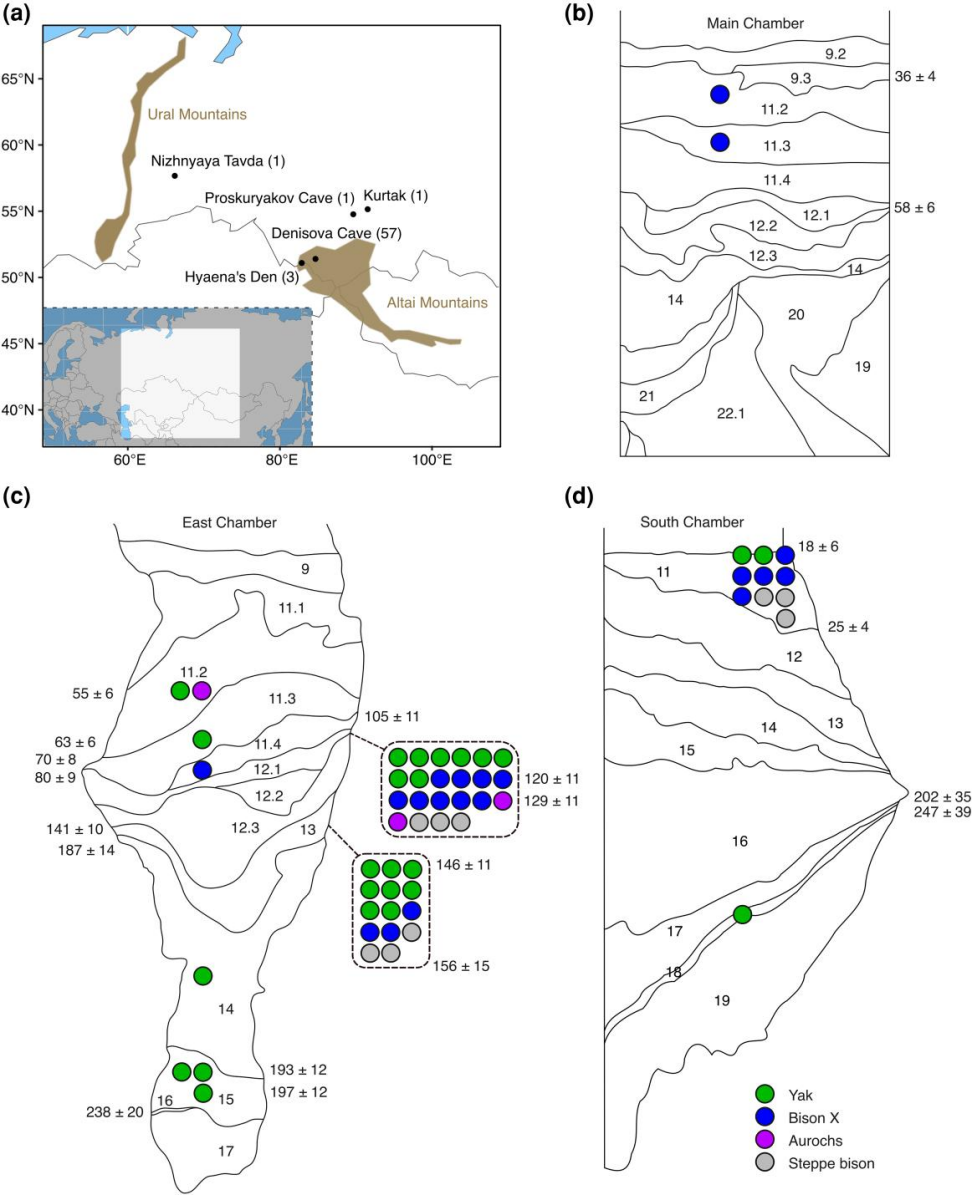
a) Map showing location of samples included in this study, number per site shown in parentheses. Denisova Cave samples with a mitochondrial depth of coverage above 0.1× are plotted in their corresponding layers from the Main Chamber (b), East Chamber (c), and South Chamber (d). Samples from layers 12 (accumulative layer of 12.1, 12.2, and 12.3) and 13 are indicated by the insets. Competitive mapping results from Table S1 are color-coded. Dates for layers including samples are shown in ka (thousand years before present, Jacobs et al. 2019, 2025). Samples from all sites other than Denisova Cave were assigned as yak.

All bovine samples presented here yielded a mitogenome depth of coverage above 0.1× and were competitively mapped for taxonomic assignment (the two best hits from the first screening panel as well as the best bovine hit are shown in Table S1). Overall, we generated a taxonomic identification for 63 bovine samples from mid-latitude Asia (Fig. 1). Three bones were identified as aurochs (*Bos primigenius*), nine as steppe bison (*Bison priscus*), and 20 as belonging to the clade X (or Bb1) bison mitochondrial lineage. Additionally, 31 samples were identified as yak (*Bos mutus*).

**Fig. 2.**
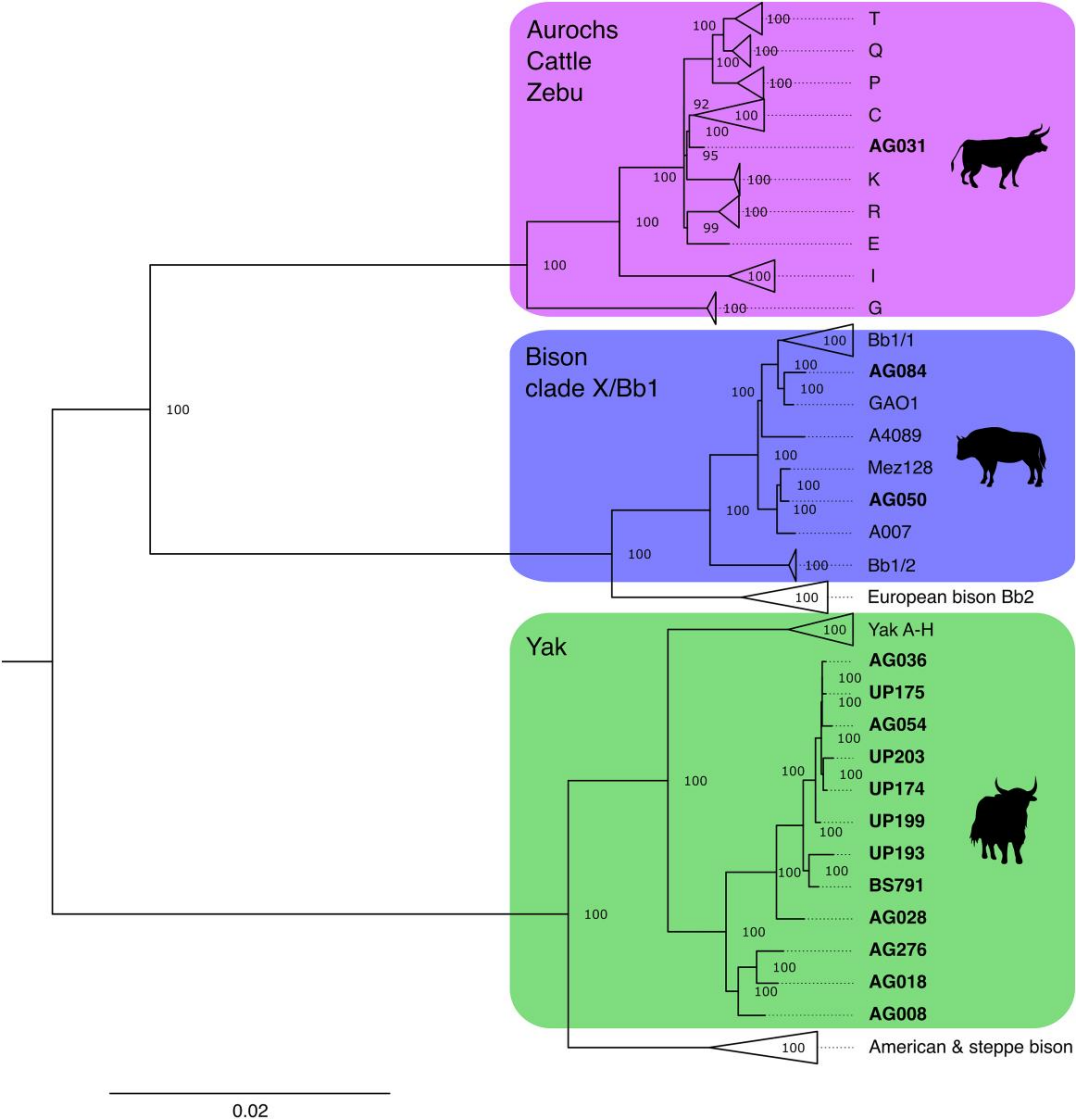
Maximum likelihood consensus phylogeny of 879 modern and ancient complete and partial bovine mitogenomes from IQ-TREE with a TPM3u + F + I + R4 model and 1,000 bootstraps based on a sequence length of 16,327 nucleotides. Node support is shown. Scale bar represents nucleotide substitutions per site. Samples generated in this study are highlighted in bold. See Table S1 (column “included in bovine phylogeny?”) and Table S5 for extensive information about sequences included. See Figs. S1 and S2 for uncollapsed versions of the aurochs C/K clades and bison clade X, respectively.

The 15 samples that yielded a mitochondrial genome with a depth of coverage above 3× after iterative mapping fell into three places within bovine mitochondrial diversity (Fig. 2, in bold), mirroring the taxonomic assignments derived from competitive mapping. This validation of our competitive mapping workflow was further investigated by downsampling these 15 samples from 1.6 – 1,463× to 0.05 – 0.56× (depth prior to iterative mapping, see Table S2). One sample identified as aurochs through competitive mapping grouped with taurine cattle (*Bos taurus*) and aurochs, specifically clustering basal to the Asian haplogroup C. Two samples clustered within the bison clade X/Bb1 lineage. Finally, the 12 samples identified as yak with competitive mapping clustered as a single clade sister to known modern and ancient yak diversity. These placements all had 100% node support based on 1,000 bootstrap replicates (Fig. 2).

### An Unknown Ancient Yak-like Mitochondrial Lineage

**Fig. 3.**
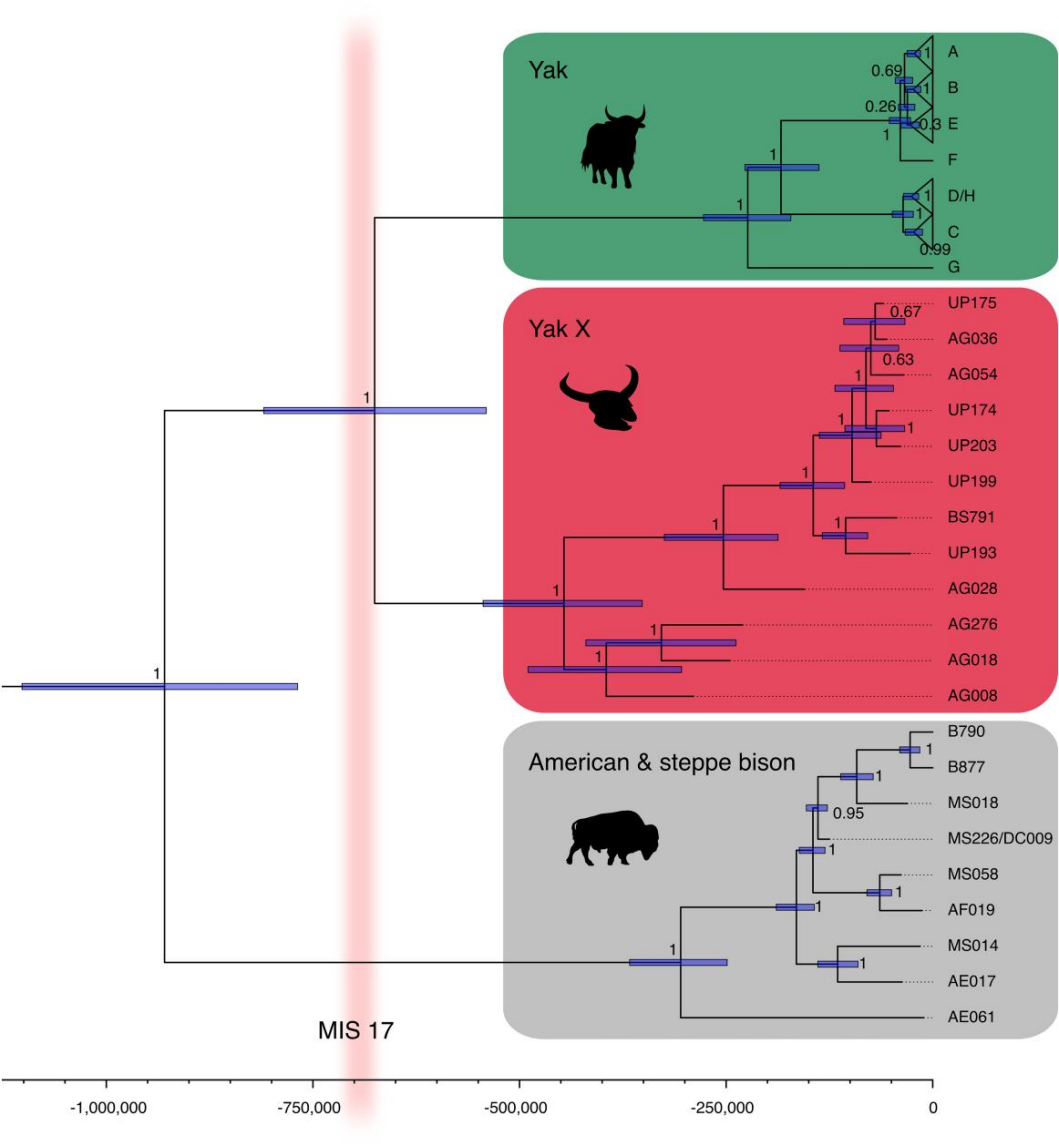
Time-calibrated BEAST mitochondrial phylogeny of modern and ancient yak rooted with American and steppe bison using a JC + G + I model and a sequence length of 15,790 nucleotides. Mean heights with 95% confidence and node posteriors are shown. Time before present is shown on the x axis in ka (thousand years before present). Known yak haplogroups are shown (see Fig. S3 for the uncollapsed phylogeny). The timing of Marine Oxygen-Isotope Stage (MIS) 17 is depicted.

Given the temporal spread of Pleistocene yak-like mitogenomes, we generated a time-calibrated phylogeny for this group (Fig. 3). In accordance with previously published phylogenies, we recovered modern-day diversity of wild and domestic yaks represented by haplogroups A to H (Ma et al. 2022). As expected, BG-67, an early Tibetan domesticate radiocarbon dated to ca. 2.4 ka (Chen et al. 2023), clustered with haplogroup A. Consistent with our bovine phylogeny presented in Fig. 2, all yak-like mitogenomes generated in this study were placed in a clade sister to known modern and ancient yak diversity.

Two out of seven samples forming the novel yak-like clade presented here successfully yielded a radiocarbon date used for calibration of our molecular clock: UP193 dated to 27,350 to 27,080 cal BP (95.4%) and BS791 dated to 44,510 to 42,710 cal BP (95.4%) (see Table S3 for details). Molecular tip-dating of 10 ancient yak-like mitogenomes belonging to this newly identified lineage show that the specimens range in age from Marine Oxygen-Isotope Stage (MIS) 8 (ca. 300 to 243 ka) to MIS 3 (ca. 57 to 29 ka) based on mean estimates (Table 1 including 95% highest posterior densities, HPD), which is roughly in agreement with the stratigraphy of Denisova Cave (Table S3). The mean estimated clock rate was 1.456 × 10^−8^ substitutions per year (ESS = 6,075, 95% HPD = [1.190 × 10^−8^ to 1.732 × 10^−8^]), roughly consistent with a previously published estimate (2.05 × 10^−8^, Zeyland et al. 2012). The split between the American/steppe bison and yak mitochondrial lineages was dated to approximately 929 ka (1,102 to 768 ka 95% HPD), whereas the split between the yak and yak-like mitochondrial lineages was dated to 675 ka (809 to 540 ka 95% HPD) (Table 2).

**Table 1:** Molecular tip-dating of yak X samples using BEAST

Sample	Mean ka	95% HPD interval (ka)	Effective sample size
AG008	290 *(329)*	385–202 *(400–236)*	4,533 (*8,143)*
AG018	245 (*338)*	338–160 *(400–256)*	4,229 (*4,963)*
AG276	230 (*274)*	321–136 *(375–167)*	4,634 (*6,910)*
AG028	155 (*226)*	229–84 *(322–135)*	5,173 (*3,063)*
UP199	75 (*114)*	118–38 *(196–32)*	964 (*1,255)*
UP175	60 (*105)*	99–22 *(177–35)*	775 (*1,029)*
AG036	56 (*105)*	96–18 *(178–36)*	791 (*1,030)*
UP174	53 (*78)*	93–16 *(147–14)*	795 (*1,371)*
UP203	39 (*51)*	78–5 *(108–0.06)*	882 (*1,719)*
AG054	35 (*116)*	72–2 *(198–35)*	1,354 (*1,366)*

Mean ka (thousand years before present) dates and 95% highest posterior densities (HPD) are derived from the 15,790-bp alignment. In parentheses, values for the 0% missingness analysis based on the 4,154-bp alignment are shown.

**Table 2:** Time to most recent common ancestor (TMRCA) for yak mitochondrial lineages estimated using BEAST

TMRCA(group)	Mean ka	95% HPD interval (ka)	ESS
TMRCA(yak A-H)	224 *(188)*	277–172 *(274–106)*	6,024 (*8,798)*
TMRCA(yak X)	446 (*440)*	544–351 *(540–343)*	3,716 (*3,931)*
TMRCA(yak A-H – yak X)	675 (*587)*	809–540 *(739–444)*	4,772 (*5,488)*
TMRCA(American/steppe bison – yak)	929 (*838)*	1,102–768 *(1039–642)*	5,875 (*7,174)*

Mean ka (thousand years before present) dates and 95% highest posterior densities (HPD) are derived from the 15,790-bp alignment. In parentheses, values for the 0% missingness analysis based on the 4,154-bp alignment are shown.

In order to account for differences in coverage depths (Table S1), and therefore missingness between yak-like mitogenomes, we performed an additional BEAST analysis with all positions containing missing data removed, yielding an alignment of 4,154 nucleotides. This resulted in systematically older tip dates (Table 1, in parentheses) and brought AG008 and AG018, the two oldest specimens recovered from the same layer, closer in age (see Fig. S4 for corresponding tree). Although we suggest coverage depth differences and missingness in our data have a minor influence on the age estimates, the chronological order of samples based on stratigraphic information remained consistent.

## Discussion

Bovines carrying a yak-like mitogenome were found to be the most abundant bovine species in the Altai mountains, closely followed by the clade X bison lineage, with only sporadic occurrences of the aurochs and steppe bison (Fig. 1). Interestingly, the frequent occurrence of high spotted hyena (*Crocuta crocuta*) hits with competitive mapping, even yielding more hits than for bovids in two cases (Table S1), is consistent with the previous suggestion that high bone fragmentation at Denisova Cave is due to predation or scavenging of bovine bones by hyenas (Brown et al. 2021). We suggest that other secondary hits to woolly rhinoceros, red deer, wolverine, or camel are due to the similarity between mammalian mitogenomes and the low mapping quality filtering applied in order to only discard perfectly multimapped reads prior to the bovine-specific competitive mapping. The relative composition of the bovine assemblage resolved by our aDNA screening was different from previous morphological and sedaDNA studies (Vasiliev et al. 2017, 2018a, 2018b; Zavala et al. 2021). Yaks have previously been considered only a rare migrant in the Altai region while the steppe bison (*Bison priscus*) was considered an abundant resident (Agadjanian and Shunkov 2018). Additionally, the more temperate aurochs has never been morphologically identified at Denisova Cave. The layers in which we recovered aurochs samples were inferred from pollen and vertebrate records to originate from warmer periods (Jacobs et al. 2019). It is worth noting that we did not obtain any higher coverage steppe bison mitogenomes (Bp, Massilani et al. 2016) to securely confirm the presence of this lineage. Out of 12 downsampled yak X samples, three generated steppe bison as the bovine hit, suggesting that such hits in our wider lower coverage dataset may also correspond to this yak-like mitochondrial lineage (Table S2).

Taken together, our results suggest that the Altai region was a hotspot for divergent and lost bovine diversity, an observation that mirrors the identification of several archaic *Homo* lineages in the region (Green et al. 2010; Reich et al. 2010; Meyer et al. 2012; Jacobs et al. 2019; Zavala et al. 2021). The samples that were confidently placed in the bovine mitochondrial phylogeny (Fig. 2) clustered within divergent extinct lineages. The bison identified in our data corresponds to the “clade X” or Bb1 lineage, which is thought to have gone extinct at the onset of the Holocene, while its sister Bb2 lineage (Massilani et al. 2016; Soubrier et al. 2016; Palacio et al. 2017) represents the extant European bison. The aurochs found in our data clustered basal to haplogroup C, the latter of which is known from ancient East and Central Asia (Zhang et al. 2013, 2023; Cai et al. 2018; Hou et al. 2024; Rossi et al. 2024). The aurochs is the wild precursor to modern cattle (*Bos taurus*) (van Vuure and van Vuure 2005). Several aurochs haplogroups survive in extant taurine breeds such as T1 to T5, P, Q, and R (Bailey et al. 1996; Troy et al. 2001; Achilli et al. 2008; Cubrik-Curik et al. 2022). The C lineage has been lost from modern mitogenome diversity, although it was identified in ancient cattle–aurochs hybrids (Chen et al. 2024). Our aurochs sample (AG031), excavated from a layer overlapping MIS 5e (Eemian; ca. 129 to 115 ka), is placed as sister to the Asian C haplogroup, which, in comparison to the divergences of other aurochs–cattle haplogroups, might represent a novel Asian aurochs haplogroup. This contributes to closing the temporal and geographic gap of a largely unsampled region, that of central Eurasia, when it comes to aurochs–cattle diversity (Sinding and Gilbert 2016).

Similarly, yak-like mitogenomes identified in the region all clustered into a previously unknown “X” lineage. This yak X lineage is sister to all previously known yak diversity (Fig. 3). Eight mitochondrial lineages (A to H) are represented in modern populations of wild and domesticated yaks (Guo et al. 2006, 2019; Wang et al. 2010, 2011, 2021, 2024; Chunnian et al. 2016; Ma et al. 2022). Although we note that the three individuals previously identified as haplogroup H (W17, XZ48, XZ67) fell within the diversity of haplogroup D (Fig. S3), this H haplogroup was previously shown to cluster in different parts of the phylogeny either basal to or nested within haplogroup D (Chen et al. 2023). In this previous study, one Late Pleistocene mitogenome represented an early yak domesticate, which clustered with haplogroup A. The yak is nowadays mainly restricted to the Himalayas but, similar to other cold-adapted species (Stewart et al. 2010), our results suggest that the yak, or a relative of it, had a wider Pleistocene distribution that probably also encompassed broader areas of Northern, Central, and Eastern Asia. This yak X lineage did not contribute to the mitogenome diversity of modern yak populations. We hypothesize that the yak X we identify genetically in the present study may correspond to the Baikal yak (*Bos mutus baikalensis*), a paleontological species identified in the Altai mountains based on earlier morphological observation (Vasiliev 2021). Further sampling of morphologically identifiable bones will be necessary to confirm this hypothesis.

All tip-calibrated dates were consistent in chronology with the stratigraphic information available, except for AG036, which appeared younger than expected (see Table S3). Although tip-calibrated dating of several samples together has been shown to cause a shift to older ages (Chacón-Duque et al. 2025), our relative dates are consistent with a long-term presence of this lineage (Table 1, Fig. 3). Direct radiocarbon dating and stratigraphy alone suggest long-term continuity of the yak X lineage in the region, from ca. 203 ka (Denisova Cave layer 15) until ca. 18 ka (layer 11). The yak X lineage thus seemingly survived in mid-latitude Asia for around 200,000 years from the Middle to Late Pleistocene, until it went extinct after the Last Glacial Maximum, following the trend of many other megafaunal species across several continents (Lorenzen et al. 2011).

We estimated the split time between the yak X and modern yak mitogenome clades to approximately 675 ka (Table 2), somewhat younger than the yak-American/steppe bison divergence time of 929 ka (Table 2), an estimate that is congruent with a previous date of ca. 820 ka (Froese et al. 2017). The former roughly corresponds to MIS 17 (ca. 712 to 676 ka), which was a particularly long interglacial (Tzedakis et al. 2022) that followed the intensification of glacial–interglacial cycles known as the Mid-Pleistocene Transition (MPT) between ca. 1.2 Ma and 800 ka (Legrain et al. 2023). It is worth noting that several other cold-adapted large mammals, including the woolly mammoth, muskox, and woolly rhinoceros, as well as Neanderthals, Denisovans, and modern humans, trace their origin to the end of the MPT (Lister 2004; Green et al. 2010; Meyer et al. 2012; Stewart et al. 2025). We therefore hypothesize that the longer and stronger climatic transitions after the MPT would have impacted cold-adapted taxa, such as yaks, by increasing the amplitude of contractions and expansions in available habitats, which in turn could have promoted population divergence (Stewart et al. 2010).

Mitochondrial and nuclear phylogenies from *Bos* and *Bison* genera present discordant topologies, which might suggest a complex evolutionary history involving processes such as ancient admixture and/or incomplete lineage sorting (Verkaar et al. 2004). The yak-like mitochondrial lineage detected in this study could therefore be carried by other bovine lineages beyond yak. Generation of nuclear genomes from yak X samples with sufficient endogenous DNA preservation (Table S1) will be necessary to further characterize this new lineage and its relationship to the yak and other bovines. While yak X possibly went extinct at the onset of the Holocene, it persisted through the last, or Eemian, interglacial. However, it is currently unclear whether the warm conditions during the Eemian interglacial (129 to 115 ka) led to a demographic bottleneck in the yak X lineage. This can be further investigated in future studies by comparing autosomal diversity in pre- and post-Eemian samples. Modern humans were shown to settle in Siberia as early as 31.6 ka BP (Sikora et al. 2019), and were also detected in sediments at Denisova Cave during the Upper Palaeolithic (Zavala et al. 2021). Yak X, and other bovines, therefore co-occured in layers corresponding to the arrival of modern humans in the Altai. Demographic analysis from nuclear genomes might help disentangle the effects of Late Pleistocene anthropogenic and climatic pressures on the extinction of yak X and other bovines, although this is still not evident for other extinct megafauna such as the woolly rhinoceros (Lord et al. 2020).

Overall, these findings demonstrate how aDNA can complement morphological and proteomics studies, enabling us to further elucidate the genetic affinities of the bovine assemblage in mid-latitude Asia during the Pleistocene. Similar to the Pleistocene hominin diversity found in the region, we identify a multitude of extinct and divergent mitochondrial lineages associated with bison, aurochs, and yaks. Our finding of a previously unknown mitogenome lineage related to the yak that probably persisted in parts of Northern, Central, and Eastern Asia for hundreds of thousands of years opens up several interesting questions, including what this taxon looked like morphologically and whether it admixed with other bovines.

## Materials and Methods

### Sample Information

We analyzed 57 bone fragments from Denisova Cave and 6 bones from other sites, all in the western and southern Siberian sector of mid-latitude Asia (Fig. 1a, Fig. S5). The Denisova Cave fragments were previously screened using ZooMS and were assigned to the Bovinae subfamily, either belonging to *Bos* or *Bison* (Brown et al. 2021). The bone fragments were recovered from layers 11.2, 11.3, 11.4, 12, 12.1 to 12.2, 13, 14 and 15 of the East Chamber, layers 11.2 and 11.3 of the Main Chamber, as well as from layers 11 and 18 from the South Chamber (Fig. 1). These layers range in age from ca. 247 to 18 ka (Jacobs et al. 2019, 2025). Site locations for the samples included in this study as well as the stratigraphic profiles from Denisova Cave are shown in Fig. 1. See Table S3 for extensive information per sample.

### Radiocarbon Dating

Between 125 and 500 mg of bone fragments were directly radiocarbon dated at the University of Vienna (Higham laboratory) or the University of California Irvine (Keck AMS facility). Radiocarbon dates were calibrated using the IntCal20 atmospheric curve (Reimer et al. 2020) with the OxCal platform v4.4.4 (Ramsey 2009). Details for the dated samples are shown in Table S3.

### Sampling, DNA Extraction, Library Preparation, and Sequencing

We followed the protocol described in Gilardet et al. (2025) to recover DNA from the Denisova Cave bone fragments. Briefly, a small piece of each sample was removed using either a cleaned circular saw attached to a Dremel tool, or with cleaned wire cutting tools. Some bone pieces were crushed to expose internal micro fractures for bleach pretreatment. This pretreatment consisted of a 4- to 15-min wash in a <0.5% sodium hypochlorite solution followed by three rounds of rinsing in double-distilled water (Boessenkool et al. 2017). Between 24 and 348 mg of bone underwent lysis following Dabney and Meyer (2019). Samples with higher amounts of bone required a longer incubation, up to ca. 4 days, in order to digest as much of the bone piece as possible. For those samples with undigested bone pellet remaining after 48 h, an additional 20 µL of Proteinase K was added. Both the low- and high-throughput DNA extraction methods described in Gilardet et al. (2025) were used in replicates for some samples (see Table S1). For the six samples from the broader locations other than Denisova Cave, ca. 250 mg of bone were pulverized in a Retsch MM400 Mixer Mill. From the resulting bone powder, 50 mg underwent bleach pretreatment as described above. DNA was then extracted following an overnight lysis using the column-based extraction protocol described in Rohland et al. (2018), with Buffer D.

Single-stranded DNA libraries were generated using the Santa Cruz Reaction protocol (Kapp et al. 2021) as modified by Nguyen et al. (2023). The libraries were then cleaned with a low- or high-throughput cleaning step depending on the extraction method followed and dual-indexed (Gilardet et al. 2025). Libraries were purified and underwent one- or two-sided size-selection with AMPure XP (Beckman Coulter) or Sera-Mag Select (Cytiva) beads, using 0.5× (for two-sided size-selection only) and 1.8× to 1.2× ratios, and then assessed with a TapeStation (Agilent) using either a D1000 or D1000-HS DNA screentape. Libraries were sequenced on either the Illumina NovaSeqX or NovaSeq 6000 platforms at the National Genomics Infrastructure (Science for Life Laboratory, Stockholm, Sweden) and the UCSF (University of California, San Francisco) Center for Advanced Technology using a 2 × 100 or 2 × 150 paired-end setup. Some libraries from three samples (AG008, AG018, and AG028) were re-amplified for 1 to 4 cycles using the KAPA HiFi kit (Roche) and primers IS5 and IS6 (Meyer and Kircher 2010) to increase the library concentration prior to further shotgun sequencing as follows: 25 µL KAPA HiFi Master Mix, 2.5 µL of each primer, 15 µL of library, to 50 µL with water.

### Data Processing, Competitive Mapping, and Mitogenome Generation

In order to generate sequencing metrics (see Table S1), raw FASTQ files were processed through the GenErode pipeline v.0.6.0 (Kutschera et al. 2022) until BAM deduplication and indel realignment, with DNA damage calculation and base quality rescaling from mapDamage activated. Default settings were otherwise applied, except for the addition of fastp (Chen et al. 2018) flags −overlap_len_require 15 and −trim_poly_g, as well as a minimum length filtering threshold of 25 bp (base pair). We used a composite reference of the bison genome assembly (*ARS-UCSC_bison1.0;* GenBank GCA_018282365.1 beefalo, USDA ARS) with a *Bison priscus* mitogenome (KX269145.1) for read mapping. We proceeded with samples that had a minimum mitochondrial depth of coverage of 0.1×. Preprocessed reads were competitively mapped in two steps. Using bwa aln v.0.7.17 and deduplication commands identical to GenErode, they were first competitively mapped against a mitochondrial reference panel including a representative of mammalian families potentially present at the sites (see Table S4). Samtools idxstats (Danecek et al. 2021) was run after filtering for multimapped reads (-q 1). The two highest hits are reported in Table S1. Then, all remaining reads aligned to the bovid bait representative (*B. taurus*) were extracted (samtools fastq -tn) and competitively mapped on a panel of wild bovines (Table S4). All higher-depth mitogenomes presented in Fig. 2 were downsampled using seqtk v1.4 to validate our competitive mapping workflow (see Table S2).

To reconstruct mitogenomes, merged and unmerged reads from preprocessed FASTQ files were iteratively mapped with the Mapping Iterative Assembler (MIA) (https://github.com/mpieva/mapping-iterative-assembler, Green et al. 2010). A *Bos mutus* (NC_025563.1), *Bison bonasus* (NC_014044.1), or *Bos primigenius* (NC_013996.1) mitochondrial reference was used as the seed, following the highest hit identified from competitive mapping. MIA was implemented with the following parameters: -c -C -U -F -k 14. An aDNA substitution matrix file was also applied using flag -s. Using the associated ma program, depth statistics were computed from the resulting maln file with -f 3 and flag -f 41 was used to generate the alternative column format file for further filtering. We used the script create_fasta_from_mia.py (https://github.com/aersoares81/mia-helper-scripts) to filter for positions with a minimum depth of 3x and 67% of agreement of the base called. For radiocarbon-dated ancient yak domesticate BG-67 (Chen et al. 2023), a consensus sequence was generated from deposited SRR files using the same methods and the *Bos mutus* reference mitogenome as seed.

### Phylogenetic Analyses

The resulting filtered consensus sequences were aligned with modern and ancient bovine mitochondrial genomes available from the literature (Table S5) using MUSCLE v.3.8.31 (Edgar 2004). All gaps and ambiguous bases were converted into missing data. The alignment was then filtered for missingness (maximum 70% per individual and 20% per site) using a script previously published in Swali et al. (2023). A bovine mitochondrial phylogenetic tree was reconstructed using IQ-TREE v.2.3.5 (Nguyen et al. 2015), using the integrated model finder to choose the substitution model (Kalyaanamoorthy et al. 2017) and 1,000 bootstraps (-m MFP -bb 1000 -alrt 1000) to evaluate branch support. The tree was visualized using FigTree v1.4.4 (https://github.com/rambaut/figtree).

We generated a similar filtered alignment comprising the yak-like mitochondrial genomes generated in this study and yak mitogenomes available from the literature (see Table S6 for mitogenomes and their ages). We included two modern American bison (*Bison bison*) and seven ancient steppe bison sequences as outgroups, which included the Ch’ijee's Bluff steppe bison dated to ca. 125 ka (Froese et al. 2017). From this alignment, we constructed a yak-specific time-calibrated mitochondrial phylogeny using BEAST v.1.10.4 (Drummond et al. 2012). We determined the best fitting model for nucleotide substitution (-s 11 -i -g 4 -f -BIC -S NNI -t ML -a) using jModelTest v.2.1.10 (Guindon and Gascuel 2003; Darriba et al. 2012). We ran BEAST twice, each for 50 million states, using the default strict clock model, a coalescent constant size tree prior, and priors on the root height (normal, mean = 8 × 10^5^, standard deviation = 1.5 × 10^5^) (Wang et al. 2018; Wu et al. 2018), clock rate (exponential, initial value = 1 × 10^−7^), and sampled ages for samples without a radiocarbon date (uniform, lower = 0, upper = 4 × 10^5^, initial = 0). Results from both runs were combined using LogCombiner after the first 10% of states from each run were removed as burn-in. TreeAnnotator was used to generate a summary tree and the output log file was examined using Tracer v.1.7.2 (Rambaut et al. 2018). Effective Sample Sizes (ESS) were checked for convergence (ESS >200). The tree was also visualized using FigTree. BEAST was additionally run on the same alignment that had been filtered for 0% missingness, using the same analysis parameters listed above.

## Supplementary Material

evaf206_Supplementary_Data

## Data Availability

Demultiplexed raw FASTQ files are available on the European Nucleotide Archive (ENA BioProject PRJEB96882). The 12 yak X and 1 aurochs mitogenome assemblies from Fig. 2 are available on GenBank with accessions PX387674 to PX387686. The script used to calculate fragment lengths is available on GitHub at https://github.com/alexandregilardet/plate_aDNA_extraction/blob/master/calculate.awk. The input xml file for BEAST is provided as a Supplementary File.
