# Supplementary material for: Paleogenomics Reveals a Loss of Bovine Lineages in Mid-latitude Asia Over the Last 200,000 Years
Source: Genome Biol Evol. 2025 Nov 6;17(11):evaf206. doi: 10.1093/gbe/evaf206 (PMC12628791; doi:10.1093/gbe/evaf206)
Supplement: evaf206_Supplementary_Data [file evaf206_supplementary_data.zip › Supporting_information_Gilardet_Oppenheimer.pdf]

Supplemental Information for:

Palaeogenomics reveals a loss of bovine lineages in mid-latitude Asia over the last 200,000 years

Alexandre Gilardet, Jonas Oppenheimer, Mikkel-Holger S. Sinding, Edana Lord, J. Camilo Chacón-Duque, Gonzalo Oteo-García, Georgios Xenikoudakis, Pavel Kosintsev, John Southon, Sergey K. Vasiliev, Michael V. Shunkov, Maxim B. Kozlikin, Katerina Douka, Beth Shapiro, Peter D. Heintzman, Love Dalén

Table of Contents:

Supplementary Figure S1: Uncollapsed aurochs clades C and K from the bovine phylogeny (Figure 2)	Page 3
Supplementary Figure S2: Uncollapsed bison clade X (Bb1) from the bovine phylogeny (Figure 2)	Page 4
Supplementary Figure S3: Uncollapsed known present-day yak section of the BEAST phylogeny (Figure 3)	Pages 5-6
Supplementary Figure S4: BEAST phylogeny with a 0% missingness threshold, using a sequence length of 4,154 nucleotides	Page 6
Supplementary Figure S5: Example photographs of several bone fragments included in this study	Page 7
Supplementary reference	Page 7
Supplementary Table S1 (excel sheet): Sequencing metrics per sample that generated a mitochondrial depth of coverage > 0.1× and was competitively mapped	

Supplementary Table S2 (excel sheet): Downsampled competitive mapping of higher mitochondrial depth of coverage samples from Figure 2	
Supplementary Table S3 (excel sheet): Sample metadata	
Supplementary Table S4 (excel sheet): Mitochondrial reference panels used for competitive mapping	
Supplementary Table S5 (excel sheet): External sequences included in the bovine phylogenetic analysis	
Supplementary Table S6 (excel sheet): External sequences used in the BEAST phylogenetic analysis	
Supplementary File: Input .xml file for BEAST	

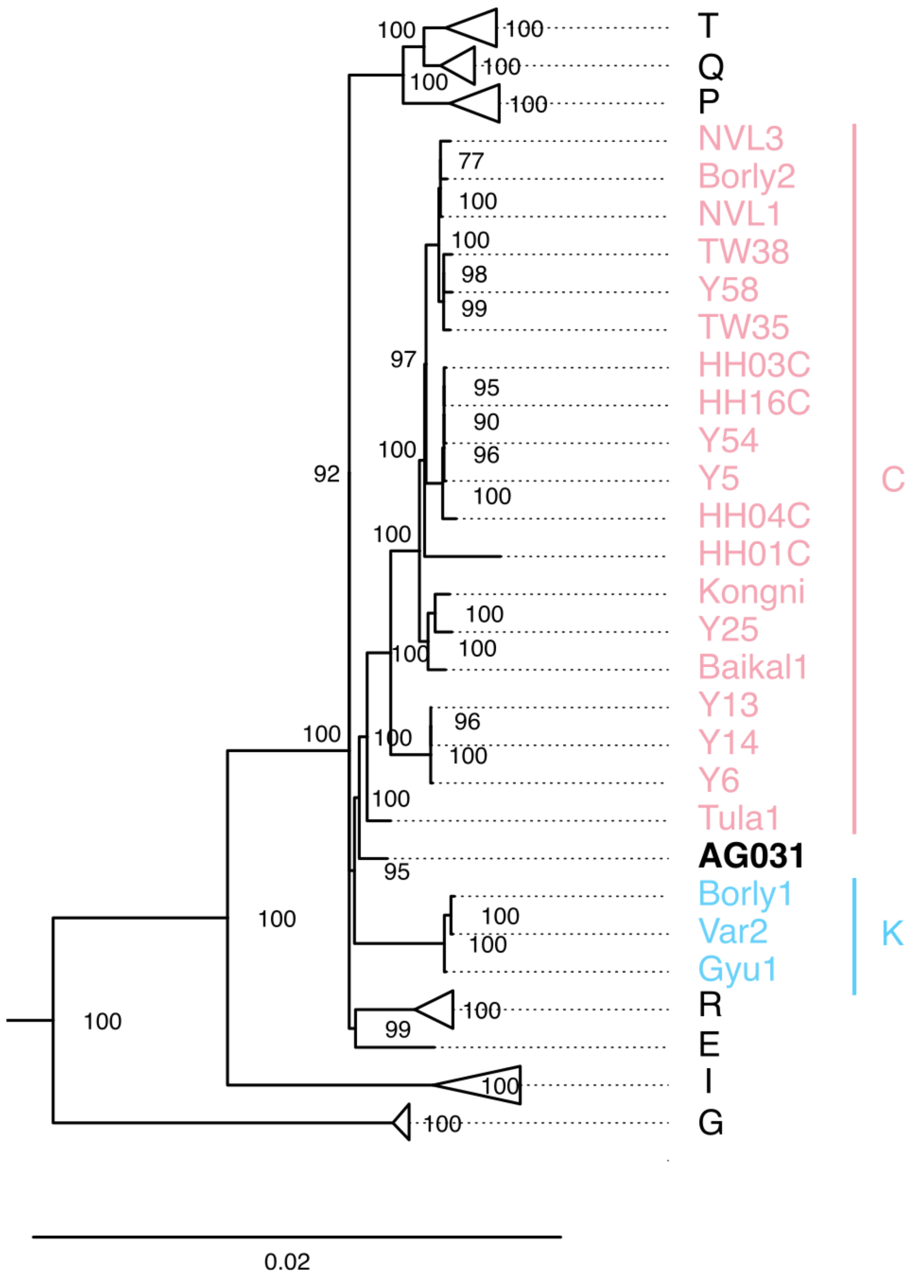

Supplementary Figure S1: Uncollapsed aurochs clades C and K from the bovine phylogeny (Figure 2). Node support is shown. Scale bar represents nucleotide substitutions per site. The sample generated in this study is highlighted in bold.

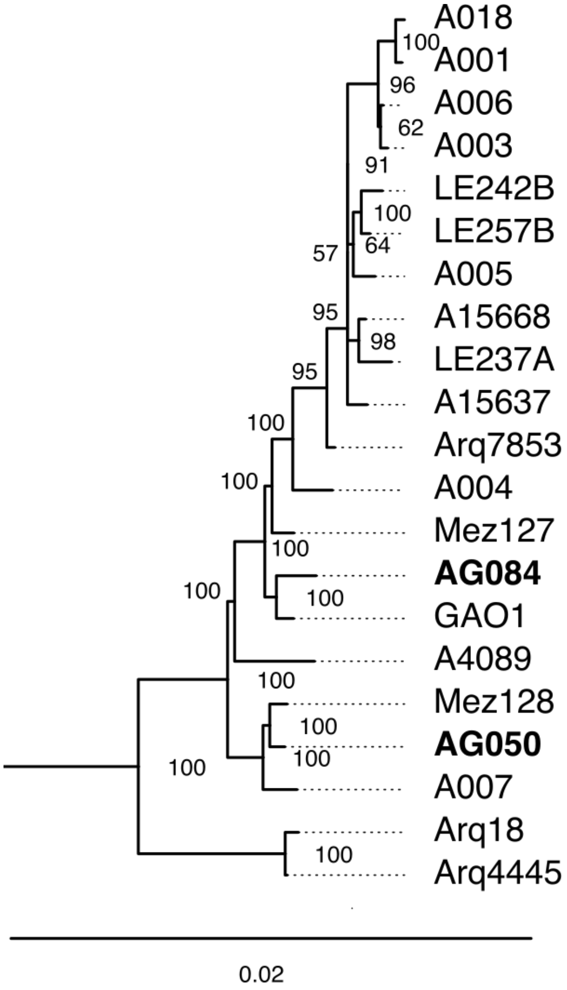

Supplementary Figure S2: Uncollapsed bison clade X (Bb1) from the bovine phylogeny (Figure 2). Node support is shown. Scale bar represents nucleotide substitutions per site. Samples generated in this study are highlighted in bold.

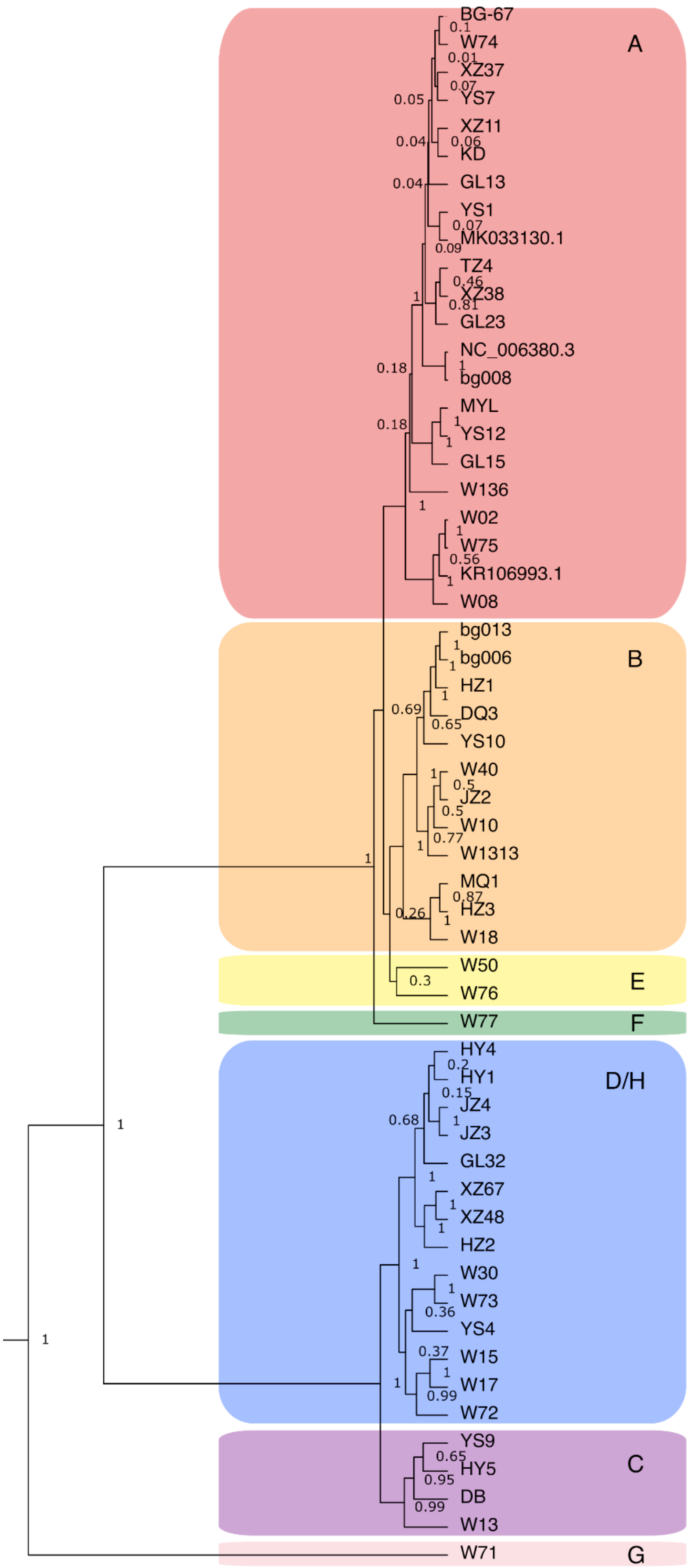
A
BG-67
W74
XZ37
YS7
XZ11
KD
GL13
YS1
MK033130.1
TZ4
XZ38
GL23
NC_006380.3
bg008
MYL
YS12
GL15
W136
W02
W75
KR106993.1
W08
B
bg013
bg006
HZ1
DQ3
YS10
W40
JZ2
W10
W1313
MQ1
HZ3
W18
E
W50
W76
F
W77
D/H
HY4
HY1
JZ4
JZ3
GL32
XZ67
XZ48
HZ2
W30
W73
YS4
W15
W17
W72
C
YS9
HY5
DB
W13
G
W71

Supplementary Figure S3: Uncollapsed known present-day yak section of the BEAST phylogeny (Figure 3). Haplogroups and node posteriors are shown.

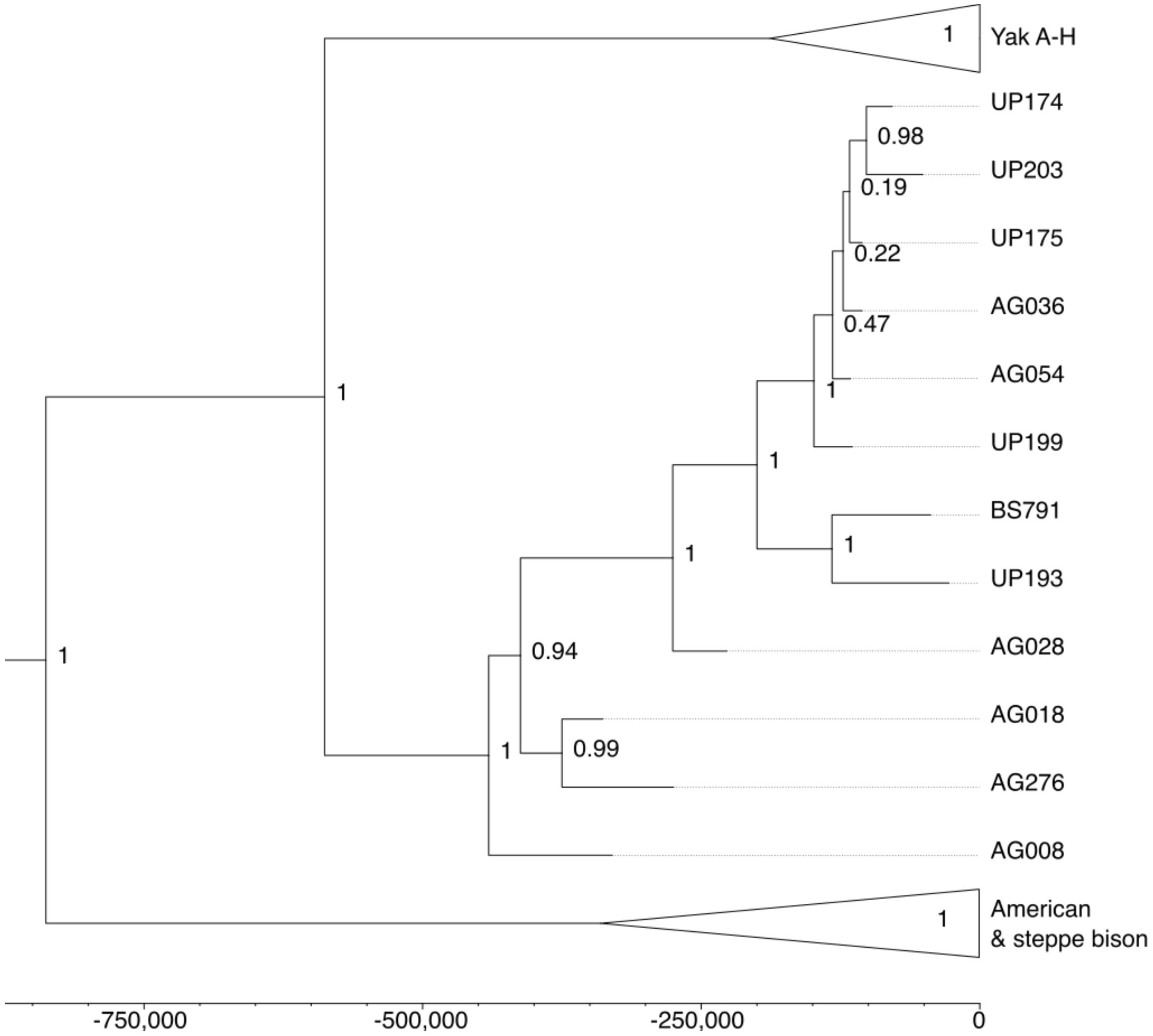

Supplementary Figure S4: BEAST phylogeny with a 0% missingness threshold, using a JC + G + I model and a sequence length of 4,154 nucleotides. Mean heights and node posteriors are shown. Time before present is shown on the x axis in ka.

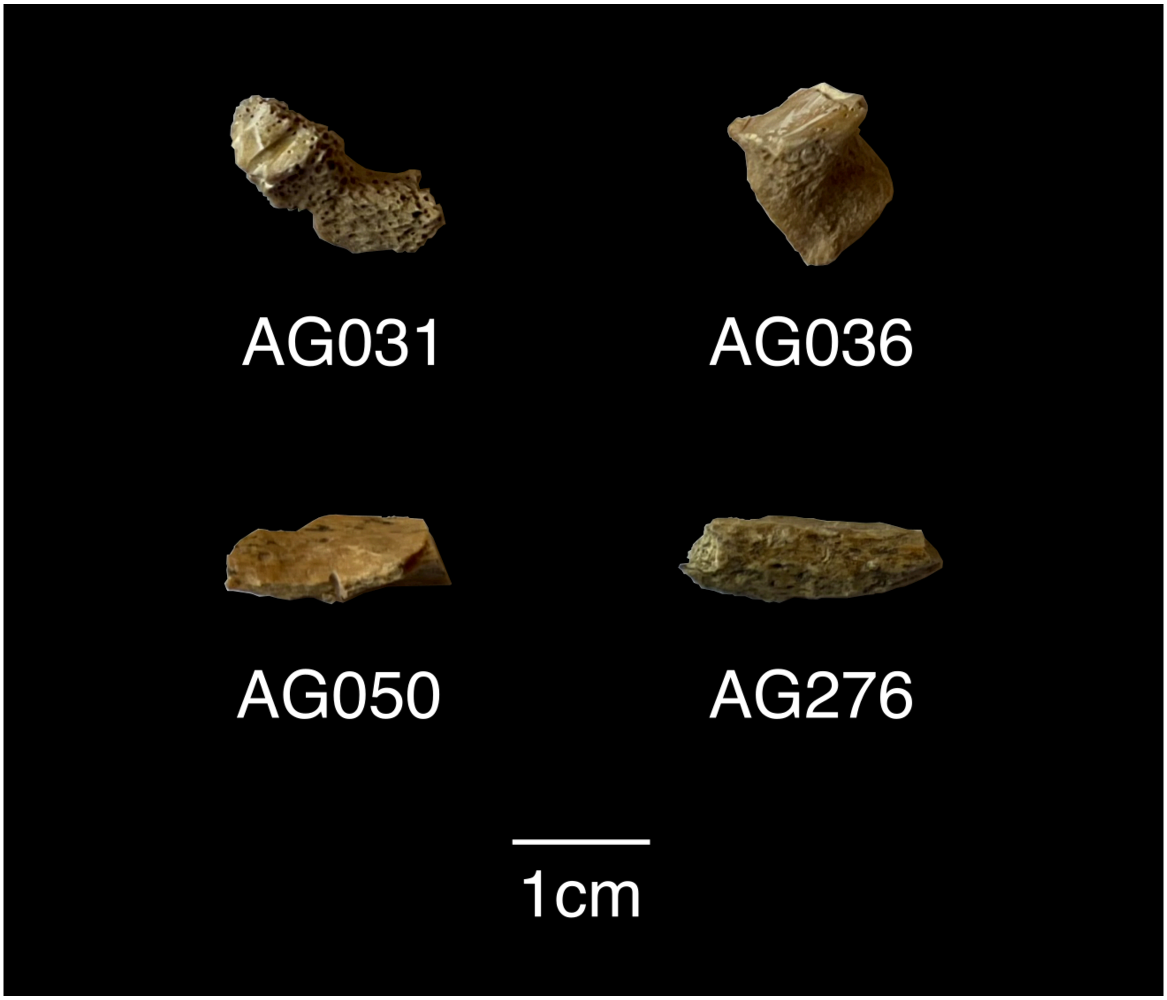

Supplementary Figure S5: Example photographs of several bone fragments included in this study. Scale is shown.

Supplementary reference

Kirillova, I.V., Chernova, O.F., Made, J. van der, Kukarskih, V.V., Shapiro, B., Plicht, J. van der, Shidlovskiy, F.K., Heintzman, P.D., Kolfschoten, T. van, Zanina, O.G., 2017. Discovery of the skull of Stephanorhinus kirchbergensis (Jäger, 1839) above the Arctic Circle. Quaternary Research 88, 537–550. https://doi.org/10.1017/qua.2017.53
